# Performance of an allele‐level multi‐locus HLA genotype imputation tool in hematopoietic stem cell donors from Quebec

**DOI:** 10.1002/iid3.185

**Published:** 2017-08-25

**Authors:** Abdelhakim Ferradji, Yasmin D'Souza, Chee Loong Saw, Karim Oualkacha, Lucie Richard, Ruth Sapir‐Pichhadze

**Affiliations:** ^1^ Research Institute McGill University Health Centre Montreal Quebec Canada; ^2^ Histocompatibility Laboratory Division of Hematology Department of Medicine McGill University Montreal Quebec Canada; ^3^ Department of Mathematics Université du Québec À Montréal Montreal Quebec Canada; ^4^ Héma‐Quebec Saint‐Laurent Quebec Canada; ^5^ Division of Nephrology Department of Medicine McGill University Montreal Quebec Canada; ^6^ Centre for Outcomes Research and Evaluation (CORE) McGill University Health Centre Montreal Quebec Canada; ^7^ Department of Epidemiology, Biostatistics and Occupational Health McGill University Montreal Quebec Canada

**Keywords:** accuracy, ambiguity, epitope, HaploStats, histocompatibility, prediction, recall, transplant

## Abstract

**Introduction:**

Donor‐recipient HLA compatibility is an important determinant of transplant outcomes. Allele‐group to allele‐level imputations help assign HLA genotypes when allele‐level genotypes are not available during donor selection.

**Methods:**

We evaluated the performance of HaploStats, an allele‐level multi‐locus HLA genotype imputation tool from the National Marrow Donor Program, in a cross‐sectional study including hematopoietic stem cell donors (HSCD) from Quebec, Canada. A total of 144 self‐identified Caucasian HSCD genotyped at the allele‐group and allele‐level for HLA‐A, ‐B, ‐C, ‐DRB1, and ‐DQB1 loci were studied. We compared allele‐level genotypes imputed by HaploStats to those obtained by the reference standard, sequenced‐based typing (SBT).

**Results:**

Imputation performance, determined by allele‐level genotype recall (fraction of matching imputed and sequenced genotypes) was 97%, 96%, 95%, 84%, and 81% for HLA‐A, ‐B, ‐C, ‐DRB1, and ‐DQB1 loci, respectively. Our sample deviated from Hardy‐Weinberg equilibrium only at the HLA‐DRB1 locus. Residual ambiguity, determined by typing resolution scores (TRS), was greatest for HLA class II loci (average TRS 0.65 and 0.80 for DRB1 and DQB1, respectively). In contrast, average TRS of 0.88, 0.84, and 0.92 was observed for HLA‐A, ‐B, and ‐C, respectively.

**Conclusions:**

HLA allele imputation from ambiguous genotypes demonstrate satisfactory prediction accuracy for HLA class I but modest prediction accuracy for HLA class II loci in self‐identified Caucasian HSCD from Quebec. While consideration of high‐resolution allele and haplotype frequencies in the Quebec population may improve the performance of available allele‐level multi‐locus genotype imputation tools in Quebec, this study suggests that genotyping at the first two field level should be conducted whenever possible.

## Introduction

Matching human leukocyte antigen (HLA) loci between donors and recipients is imperative to minimize immune‐mediated injuries following solid organ transplantation 1 and hematopoietic stem cell transplantation 2. Accurate HLA genotype prediction is of particular importance in the field of histocompatibility considering the transition toward epitope‐based donor‐recipient compatibility evaluation 3, 4, 5. Assessing HLA compatibility at the allele‐level for the HLA‐A, ‐B, ‐DRB1, and ‐DQB1 loci as well as the HLA‐C, ‐DRB3/4/5, ‐DQA1, and ‐DPA1/B1 loci has been proposed as a strategy to prevent the development of donor‐specific anti‐HLA antibodies (DSA), which are deleterious to the transplanted organ 1.

Allele‐level typing by next‐generation sequencing (NGS) is rapidly becoming the standard of care in hematopoietic stem cell transplantation 6. NGS is likely to be conducted when new donors are recruited to stem cell registries. However, in the case of legacy hematopoietic stem cell donors (HSCD) who have been in stem cell registries for some years and recruitment typing laboratories that have not yet adopted NGS technology or high‐throughput automated SBT technology, reanalysis of HSCDs’ HLA types across registries by NGS would represent an unrealistic endeavor. Consequently, in the foreseeable future, the readily available methods for HLA genotyping of unrelated HSCD will continue to yield results with allelic and phase ambiguity and/or incomplete representation of all clinically relevant loci 1, 7. Furthermore, in solid organ transplantation, cost and time restrictions often make allele‐level HLA genotyping impractical. Consequently, only intermediate‐ or even low‐resolution typing may be available at the time of deceased donor organ allocation.

To overcome the limited availability of high‐resolution HLA typing, low‐to‐high‐resolution imputation tools are often used to estimate allele‐level donor and recipient HLA types 7. Such methods are applied in an effort to decrease the ambiguity and, therefore, increase the resolution of HLA data and facilitate rapid identification of compatible donor recipient pairs with minimal additional costs. In Quebec, like many other jurisdictions across the world, high‐resolution HLA types of the entire population are not readily available and HaploStats, a web‐based application provided by the U.S. National Marrow Donor Program (NMDP) Bioinformatics Group for imputation of high resolution HLA genotypes from multi‐locus unphased genotypes (http://www.haplostats.org), is often used in clinical practice to predict allele‐level genotypes. Because haplotype frequencies may vary across populations, we conducted a cross‐sectional study to investigate the performance of HaploStats in the Quebec population. This validation was performed using a convenience sample of hematopoietic stem cell donors (HSCD) selected for transplantation for whom both ambiguous first field genotypes and unambiguous first two field genotypes were available.

## Materials and Methods

### Study design and ethics statement

The McGill University Health Center Research Ethics Board approved this cross‐sectional study and it is reported in accordance with the STROBE initiative. All volunteer donors from the Héma‐Quebec registry signed an informed consent form stating that their HLA data can be used for genetic research.

### Data set and HLA typing formats

The Héma‐Quebec registry includes donors who are typed at the HLA‐A, ‐B, ‐C, and ‐DRB1 loci with a proportion of donors typed only at the HLA‐A, ‐B and ‐DRB1 loci decreasing over time. In all participants, HLA types are obtained by molecular methods. At the time of recruitment, ambiguous first field (see http://hla.alleles.org/nomenclature/naming.html) HLA types at HLA‐ A, ‐B, ‐C, and ‐DRB1 loci are obtained from Dynal reli SSO typing kits from Invitrogen‐Life Technologies (Dynal Biotech Ltd., Bromborough, Wirral, UK) or Lifecode Luminex SSO typing kits from Immucor (Immucor Transplant Diagnostics Inc., Stamford, CT). Once selected for donation, first two field HLA‐A, ‐B, ‐C, ‐DRB1, and ‐DQB1 types are obtained using SBT Celera (Alameda, CA) sequencing kits from Abbott Molecular (Abbott Laboratories Ltd., Mississauga, ON, Canada).

The analytic data set consisted of HLA typings from a total of 176 HSCD who were selected for donation and included in the Héma‐Quebec registry of HSCD. The self‐identified ethnicity of most donors was Caucasian (*n* = 144). The remaining donors were Asian (*n* = 7), Black Canadians (*n* = 2), Hispanics (*n* = 2), and 21 were of unknown ethnicity. Considering the small number of non‐Caucasian donors in this sample, the imputation performance of HaploStats was evaluated only in the Caucasian subgroup.

Molecular typings were available at mixed levels of resolution, with alleles reported at the first field level, and ambiguities listed as NMDP codes (https://bioinformatics.bethematchclinical.org/hla/alpha.v3.html), or at the first two field level. When only first two field types were available (HLA‐DQB1), ambiguities were introduced into the data by masking allele types to the first field allele‐group level.

### Imputation method

To validate allele‐level (genotype) imputation by HaploStats, we compared imputed HLA classes I and II allele types to those obtained by molecular methods. Ambiguous first field HLA types were entered into HaploStats (http://www.haplostats.org) and first two field HLA types were imputed using the NMDP full 2011 HLA Dataset for Caucasians. HaploStats considers HLA haplotype frequency information relative to various global, country and ethnically specific populations. Using these haplotype frequencies and assuming Hardy–Weinberg equilibrium (HWE), the HaploStats algorithm considers all the possible phased genotypes for the ambiguous HLA types to calculate probabilities of the most likely phased genotypes 7. HaploStats also computes the most likely unphased genotypes using genotype frequencies obtained by summing all phased genotype frequencies so that pairs of haplotypes are converted into unphased genotypes 8. As part of the imputation process, the highest likelihood predicted allele‐level multi‐locus HLA genotype was assigned and evaluated. Prior to evaluating the performance of the imputation tools, alleles in the unambiguous and imputed datasets were standardized by “G” groups (http://hla.alleles.org/wmda/hla_nom_g.txt). G groups represent a selection of HLA alleles with identical nucleotide sequences across the exons encoding the peptide‐binding domains (exons 2 and 3 for HLA class I and exon 2 for HLA class II alleles). Standardization by G groups prevents underestimation of the imputation performance by HaploStats as a consequence of discrepancies between imputed allele‐level HLA types and reference HLA types belonging to the same G group.

### Validation of first field to first two field HLA type imputation by HaploStats

#### Imputation accuracy

To evaluate imputation accuracy, we calculated the weighted city block distance and recall. Weighted city block distance evaluates the proximity between the observed and expected proportion of correctly imputed allele‐level multi‐locus genotype results (*d_w_*,Supplementary Material 1, equation  1) 9. Recall is defined as the fraction of correct HLA types (i.e., matching the true HLA types as determined by SBT that were predicted by the imputation algorithm in the tested ambiguous dataset (Supplementary Material 1, equation  2).

#### Imputed genotype ambiguity measures

We used a typing resolution score (TRS) to quantify the uncertainty (i.e., ambiguity) associated with the genotype imputation process (Supplementary Material 1, equation  3). The TRS score is bounded by 0 and 1 with low scores indicating highly ambiguous imputation results and high scores indicating little residual ambiguity 10.

#### Verification of Hardy–Weinberg equilibrium (HWE)

Verification of HWE on HLA data was conducted using a nested likelihood ratio test. This test is included among the computer tools UNIFORMAT.v3 and GENE[RATE], which have been adapted to analyze ambiguous nominal data in population genetics 11.

#### Comparison of sample alleles and haplotypes with referenced allele and haplotype frequencies

To ensure our sample is representative of the Quebec HSCD population, we compared the distribution of allele frequencies in our sample (*n* = 144) to those reported by Buhler et al. 12 for allele groups at the HLA‐A, ‐B, and ‐DRB1 loci using the equality proportion test 13. We then evaluated for the presence of rare alleles. Finally to assess whether the performance of HaploStats was related to presence, in our study sample, of new haplotypes that were not previously reported, we first delineated all possible haplotype pairs, given the genotypes measured for each participant and then verified whether these haplotypes were also reported in the US NMDP, the Registre France Greffe Moelle (RFGM) 14 and/or the Allele Frequencies website (http://www.allelefrequencies.net). The computer tools UNIFORMAT.v3 and GENE[RATE] were used to estimate haplotype and allele (at the first two field level) frequency distributions by HLA‐locus based on the expectation‐maximization (EM) algorithm 11.

## Results

### Imputation accuracy

#### Weighted city block distance

**Figure 1:**
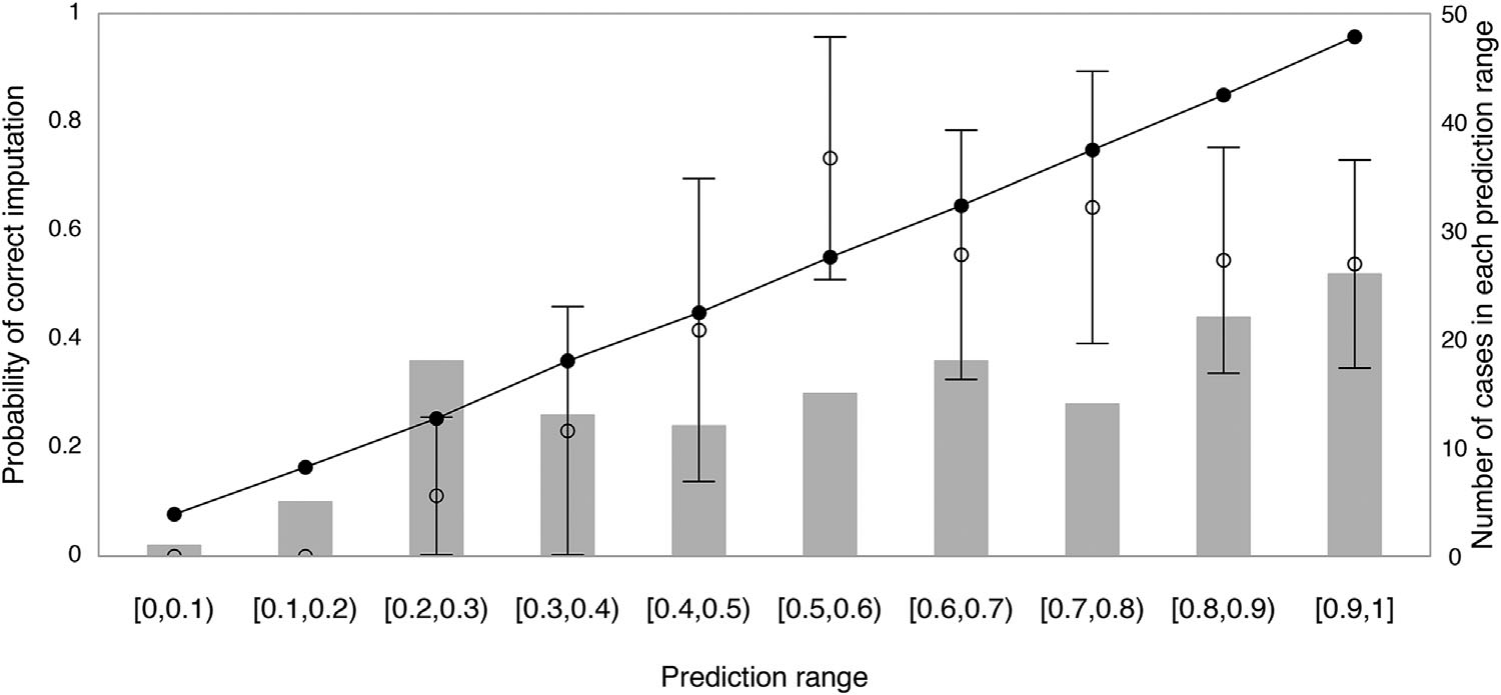
Validation of imputation accuracy at the unphased multi‐locus genotype level. The prediction interval [0, 1] was subdivided into 10 bins. The proportion of correctly imputed genotypes and their 95% confidence bounds were calculated for each interval using normal approximation. The number of total predictions in each interval is represented by vertical bars. In the interval [0.4, 0.5], for example, 12 imputed genotypes were present whose imputation probability was between 0.4 and 0.5. The observed fraction of correct imputation was 5/12 = 0.42 (○), while the expected fraction of correct imputations measured by the average predicted probability in this bin was 0.44 (•).

The weighted city block distance results for HLA classes I and II multi‐locus unphased genotypes are presented in Figure 1. This figure demonstrates the observed proportions of correct imputations and the 95% confidence bounds for each interval. The prediction interval [0, 1] was subdivided into 10 bins. The total predictions in each bin are represented by vertical bars. For the lower prediction ranges (bin 1 and 2) confidence intervals could not be displayed since the observed correct prediction was zero and confidence intervals could not be calculated by normal approximation. In the other bins, larger confidence interval bounds were observed as a consequence of the relatively small number of participants represented in each bin. In the intermediate bins (bins 3–8), the expected correct predictions fall, in general, within the bounds of the confidence intervals; however, the distance between observed and expected probabilities appears greater. Surprisingly, whereas a high correct imputation percentage should be observed in the higher prediction ranges (bins 9 and 10), we notice that the imputed correct fractions were below expected.

Table 1 summarizes weighted city block distance values when considering individual HLA loci as well as aggregates by HLA class. Observed predictions appear to align well with expected proportions, resulting in a smaller distance for class I loci. Class II loci, in contrast, deviate from the expected proportions as reflected by greater observed distances.

**Table 1:** Weighted city block distance and average typing resolution score across HLA loci

HLA Locus	*d_w_*	Average TRS
A∼B∼C∼DRB1∼DQB1	0.22	0.49
A∼B∼C	0.07	0.73
DRB1∼DQB1	0.22	0.62
A	0.02	0.88
B	0.05	0.84
C	0.04	0.92
DRB1	0.06	0.65
DQB1	0.18	0.80

*d_w_*, weighted city block distance; TRS, typing resolution scores.

### Recall

**Figure 2:**
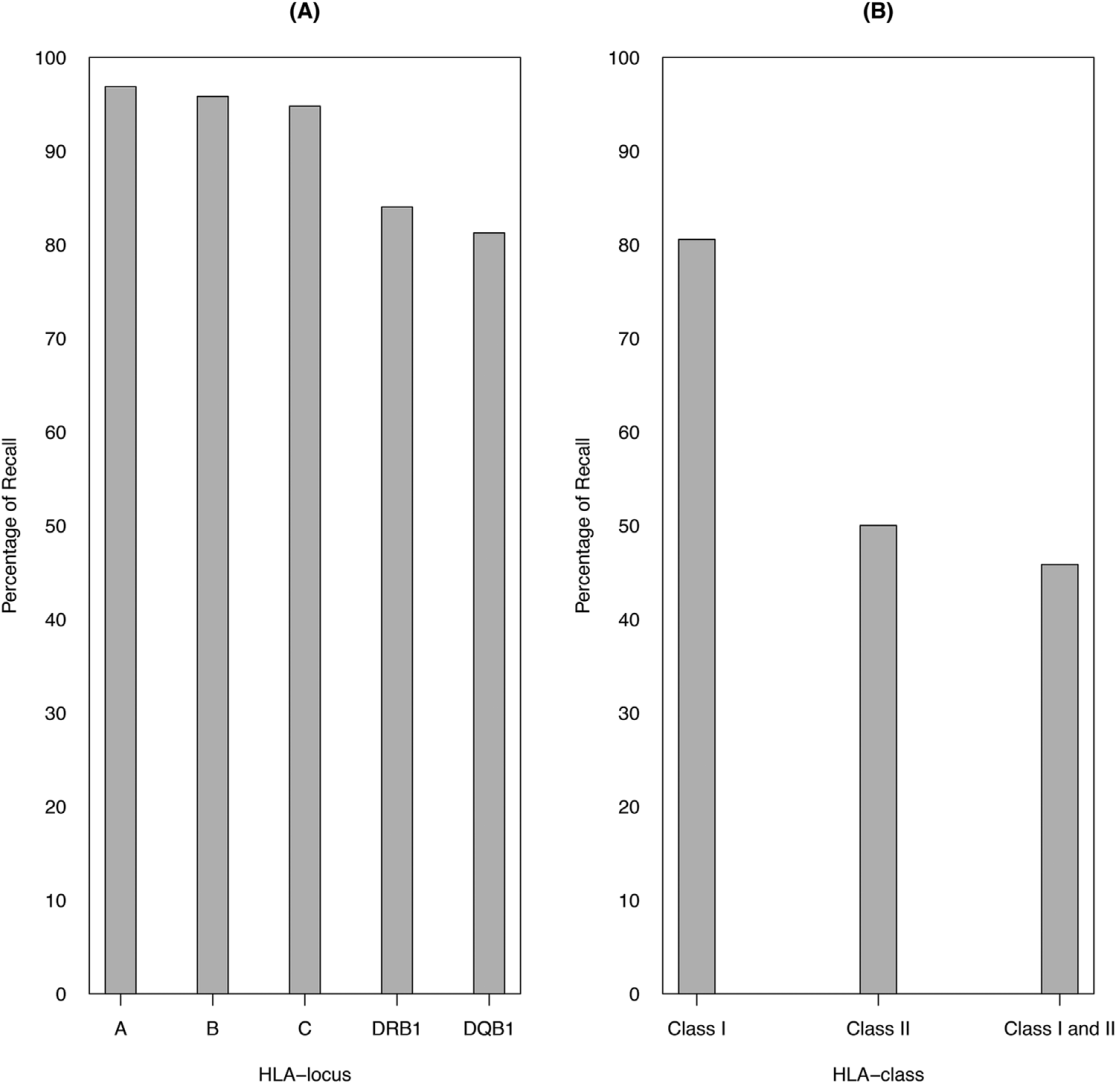
Average percent recall by HLA locus and class. Average percent recall is defined as the fraction of correctly imputed unphased HLA genotypes (i.e., matching the genotypes determined by sequence‐based typing) that were predicted by the imputation algorithm in the tested ambiguous Héma‐Quebec dataset. On the left (A), results are presented per HLA locus. On the right (B), results are presented by class (I or II) and across loci (classes I and II).

Allele‐level, HLA class‐level, and multi‐locus‐level genotype percent recall values are presented in Figure 2. On average, allele‐level genotype recall was 97%, 96%, 95%, 84%, and 81% for HLA‐A, ‐B, ‐C, ‐DRB1, and ‐DQB1 loci, respectively. When considering recall by HLA class, recall values of 80% and 50% were observed for the HLA classes I and II loci, respectively. Allele‐level unphased genotype recall across all loci (HLA classes I and II) was 46%.

### Imputed genotype ambiguity measures

#### Typing resolution score (TRS)

**Figure 3:**
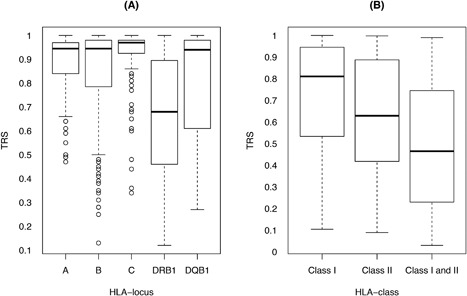
Distribution of typing resolution score by HLA locus and class. Distribution of typing resolution score of unphased genotypes for HLA‐A, ‐B, ‐C, ‐DRB1, and ‐DQB1 loci in the Héma‐Quebec dataset is presented. On the left (A), results are presented per HLA locus. On the right (B), results are presented by class (I or II) and across loci (classes I and II).

Figure 3A shows TRS distribution by HLA locus. Average TRS of 0.88, 0.84, and 0.92 were observed for HLA‐A, ‐B, and ‐C loci, respectively. In the case of HLA‐DRB1 and the HLA‐DQB1 loci, average TRS of 0.65 and 0.80, respectively, were observed. When considering TRS distribution for each unphased genotype by HLA class (Fig. 3B), we observed greater uncertainty in the imputation process. This was most pronounced in the case of HLA‐DRB1, and, consequently, HLA class II loci (average TRS 0.65).

### Verification of HWE

The nested likelihood ratio test demonstrated no deviation from HWE at the HLA‐A, ‐B, ‐C, and ‐DQB1 loci (*P*‐value = 1 for HLA‐A, ‐B, and ‐C loci and *P*‐value = 0.159 for HLA‐DQB1 locus). In contrast, the HLA‐DRB1 locus in our study sample was found to deviate from HWE (*P*‐value = 0.021).

### Comparison of sample alleles and haplotypes with referenced allele and haplotype frequencies

**Figure 4:**
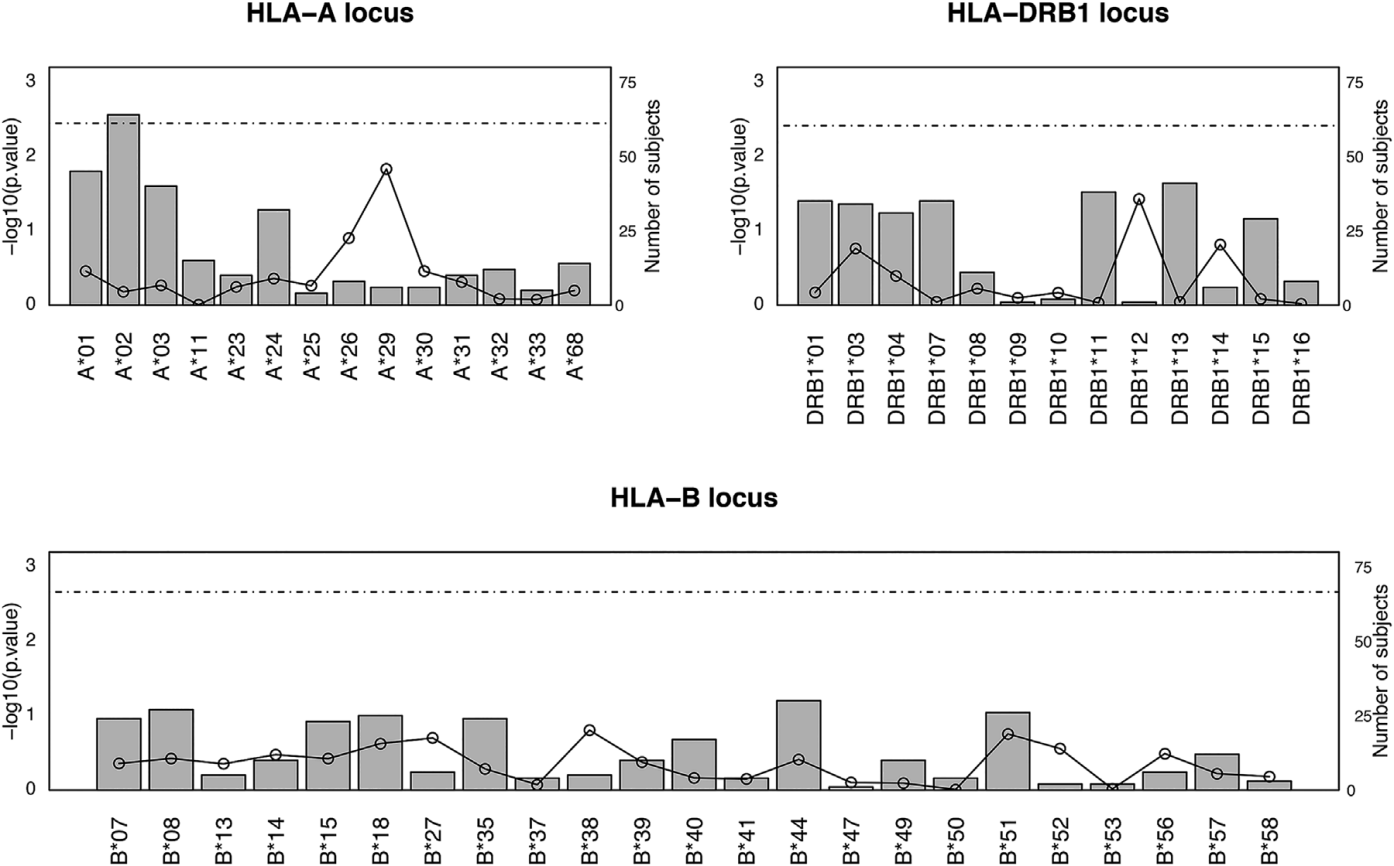
Equality proportion test results for HLA‐A, ‐B, and ‐DRB1 loci. The proportion equality test‐statistic was used to evaluate whether the frequencies of first field HLA‐A, ‐B, and ‐DRB1 types observed in the study sample are comparable to the larger Quebecer hematopoietic stem cell donor population reported by Buhler et al. 12. *P*‐values of the equality test‐statistic in a −log_10_ scale are plotted for each locus. The threshold of significance following a Bonferroni correction for multiple testing is represented by the dotted horizontal line. The number of subjects (from a total study sample of 144 participants) with each HLA type is represented by vertical bars.

When verifying whether our sample was representative of the Quebec population of HSCD, the equality proportion test was used to compare the allele‐group distributions in our sample with those previously reported across Quebec HSCD 12. This analysis demonstrated a similar distribution of alleles at the HLA‐A, ‐B, and ‐DRB1 loci in the two datasets (see Fig. 4). Allele and first two field haplotype frequencies in our study sample are presented in Supplementary Material 2.

When assessing impact of rare alleles on the performance of allele‐level multi‐locus genotype imputation by HaploStats using the Héma‐Quebec dataset, we found that from a total of 144 self‐reported Caucasian hematopoietic stem cell donors (HSCD), nine (6.25%) demonstrated rare alleles (see Supplementary Material 3). In the case of 88% of the genotypes in our study sample, at least one of the possible haplotype pairs was reported previously. These explained a total of 42% of the inaccurately predicted HLA genotypes. In the case of the remaining (12%) genotypes, only one or none of the haplotypes from each possible haplotype pair was reported previously. All these genotypes were, consequently, incorrectly imputed (see Supplementary Material 4).

## Discussion

We evaluated the performance of HaploStats, an allele‐level multi‐locus imputation tool, in a convenience sample of 144 self‐identified Caucasian HSCD from Quebec. Our analyses found that imputation by HaploStats demonstrates satisfactory HLA type prediction accuracy for class I loci but only moderate HLA type prediction accuracy for class II loci. Moreover, loci demonstrating lower HLA type prediction accuracy, expressed as lower percent recall and greater weighted city block distance, exhibit greater ambiguity.

To our knowledge, this study is the first to evaluate the performance of the HaploStats imputation tool in an independent sample. In Quebec, like many other jurisdictions, population‐level allele frequencies are not readily available. While allele‐level typing by NGS is rapidly becoming the standard of care in hematopoietic stem cell transplantation 6, in clinical practice at the time of solid organ allocation, HaploStats is often used to impute allele‐level genotypes. On average, allele‐level recall values of 97%, 96%, and 95% were observed for HLA‐A, ‐B, and ‐C loci, respectively. These results are reinforced by proximity between the correctly observed and expected proportions of correct imputations using the weighted city block distance measure for HLA class I (Table 1). In contrast, on average, an allele‐level genotype recall values of 84% and 81% were observed for HLA‐DRB1 and ‐DQB1, respectively. The modest average recall observed for class II loci may be explained by several factors.

First, reliance on a self‐reported ethnicity imposes the assumption that individuals belong to a single ethnicity and that the self‐reported ethnicity is accurate. Ethnicity categories currently considered in the Héma‐Quebec 12 recruitment questionnaires and the NMDP registry 8, on which HaploStats bases its algorithm, do not account for individuals listing more than one race or ethnic background, despite the likelihood of multi‐ethnic descent among both United States and Quebecer HSCD. Moreover, genomic and statistical evidence show that while 5–15% of genetic variation occurs between large groups living on different continents, the remaining majority of variation occurs within such groups 15, 16, 17. Such ancestral discrepancies may, consequently, contribute to inaccuracies when imputing HLA types when allelic distributions in the population are unknown.

Second, haplotype frequency estimations used by HaploStats when calculating imputation probabilities rely on HWE. Our sample deviated from HWE for the DRB1 locus. Interestingly, when using low‐resolution HLA‐A, ‐B, and ‐DRB1 types from the Quebec HSCD registry in an era preceding the current study sample, Buhler et al. 12 demonstrated that donors from most Quebec regions were in HWE except for those from the Montreal region 12. Donors from the Montreal region tended to deviate from HWE at HLA‐B and ‐DRB1 loci. This was related to a high level of genetic diversity in this region because of the substantially high immigration rates in the Montreal and Laval areas and higher rates of donor recruitment from Quebec's most highly populated areas.

Third, in addition to limitations arising from the reliance of registry analyses on single self‐identified ethnicity, potential ancestral discrepancies, and deviation from HWE, the modest number of HSCD who had both first field and first two field HLA types may also contribute the underestimated prediction probabilities (Fig. 1) in the low and high prediction range bins of weighted city block distance results. The accuracy of computationally inferred haplotypes increases with sample size, even with the use of samples from multiple ethnicities 18, 19. Despite this, it can be argued that, at a minimum, our findings represent the performance of HaploStats in both our sample of HSCD and their recipients. Furthermore, we found first field HLA frequencies to be consistent between our sample and those of the larger population of HSCD previously reported by Buhler et al. 12, with the exception of C and DQB1 loci, which are missing from Buhler's study. This suggests that our observations on the performance of HaploStats may be applicable across HSCD registered in Quebec including those who were not selected for donation.

Fourth, the performance of HaploStats is evaluated against the recently used reference standard for first two field HSCD HLA genotyping (i.e., SBT) in Quebec. This reference standard, however, is vulnerable to residual genotype ambiguity. While HLA typing of HSCD is transitioning to NGS, continued use of HaploStats, for example, in the setting of solid organ transplantation, warrants re‐evaluation of its performance in a larger external population‐based random sample that is genotyped using NGS. The latter method may also be less prone to under‐representation of rar(er) phenotypes. It is important to note, however, that only a minority (9/144) of study participants demonstrate one or more rare alleles (see Supplementary Material 2). Continued use of imputation tools like HaploStats to identify compatible HSCD from the registry or at the time of deceased donor allocation for solid organ transplantation, warrants consideration of HLA haplotype and allele frequencies in the larger Quebec population. High‐resolution typing at the population level may also be less prone to under‐representation of rar(er) phenotypes.

For completeness, it is important to note that additional population‐specific algorithms are currently available for the identification of compatible HSCD. These tools impute first two field HLA types and rely on intermediate‐ or high‐resolution HLA types in European populations. OptiMatch 20 and EasyMatch 21 account for linkage disequilibrium between alleles of adjacent loci as well as haplotype frequencies in their respective populations to assign the most likely haplotype given ambiguous allele pairs. Imputed HLA types are then used to rank HSCD per their compatibility with transplant candidates. OptiMatch facilitates accurate imputation of donor genotypes at HLA‐A, ‐B, ‐C, ‐DRB1, and ‐DQB1 loci thanks to the availability of 145,000 intermediate‐ or high‐resolution haplotype frequencies of active donors of the German Zentrales Knochenmarkspender‐Register while EasyMatch relies on high‐resolution haplotype frequencies from 42,636 unrelated donors registered in the RFGM in addition to published haplotype frequencies in various ancestry backgrounds. EasyMatch further computes the expected probabilities of identifying a compatible donor within the available pool. Because approximately 90% of the gene pool in Quebec is considered to have originated from French founders 22, it may seem appropriate to consider French haplotype frequencies when imputing high‐resolution HLA types in this population. The analysis of our sample, however, finds haplotypes that were neither reported in the US NMDP nor in the RFGM (Supplementary Material 4). This observation and a previously reported higher genetic variation across Quebec (Fixation Indices [FSTs] between 0.16% and 0.32%) in reference to that observed in France (FSTs not significantly different from 0) 12, can be explained by subdivision of privileged population secondary to the colonization of distant regions by multiple and successive founder effects 23, 24. These findings suggest that consideration of French haplotype frequencies will also be insufficient to overcome inaccurate first two field genotype predictions in the Quebec population.

In summary, while HaploStats may be used for imputing allele‐level HLA class I types in Caucasian Quebecer HSCD, it may contribute to greater misclassification of class II first two field HLA types. The growing interest in evaluating donor‐recipient compatibility at the epitope level and their role in predicting transplant outcomes make it imperative to ensure accurate allele‐level HLA type assignment 25, 26, 27, 28. Despite the undeniable advantages of imputation tools, including the ability to decrease ambiguity at low cost and a quick turnaround time in comparison to resource‐intensive DNA‐based typing methods 7, our findings suggest that genotyping at the first two field level should be conducted whenever possible.

## Authors' Contributions

Study concept and design: KO, CLS, RSP. Acquisition of data: LR, RSP. Analysis and interpretation of data: AF, YD, CLS, KO, LR, RSP. Drafting of manuscript: AF, YD, CLS, KO, LR, RSP. Data management and statistical analyses: AF, YD, CLS, KO, LR, RSP. Critical revision of manuscript: AF, YD, CLS, LR, KO, RSP.

## Consent for Publication

Each author contributed important intellectual content during manuscript drafting or revision and accepts accountability for the overall work by ensuring that questions pertaining to the accuracy or integrity of any portion of the work are appropriately investigated and resolved.

## Conflict of Interest

None declared.

## Supporting information

Additional supporting information may be found in the online version of this article at the publisher's web‐site.

Supporting Information S1.

Supporting Information S1.
