# Supplementary material for: Performance of an allele‐level multi‐locus HLA genotype imputation tool in hematopoietic stem cell donors from Quebec
Source: Immun Inflamm Dis. 2017 Aug 25;5(4):551–9. doi: 10.1002/iid3.185 (PMC5691302; doi:10.1002/iid3.185)
Supplement: Supplementary file 1 — Supporting Information S1. [file IID3-5-551-s001.dotm]

STROBE Statement—Checklist of items that should be included in reports of ***cross-sectional studies***

|  | Item No | Recommendation |
| --- | --- | --- |
| **Title and abstract** | 1 | (*a*) Indicated the study’s design in the abstract |
|  |  | (*b*) Provided in the abstract an informative and balanced summary of what was done and what was found |
| Introduction | | |
| Background/rationale | 2 | Explained the scientific background and rationale for the investigation being reported |
| Objectives | 3 | Stated specific objectives, including any prespecified hypotheses |
| Methods | | |
| Study design | 4 | Presented key elements of study design early in the paper |
| Setting | 5 | Described the setting, locations, and relevant dates, including periods of recruitment, exposure, follow-up, and data collection |
| Participants | 6 | (*a*) Gave the eligibility criteria, and the sources and methods of selection of participants (See “Study design, ethics statement, data set, and HLA typings formats” section) |
| Variables | 7 | Clearly defined all first two field HLA genotype imputation performance parameters |
| Data sources/ measurement | 8* | For each variable of interest, gave sources of data and details of methods of assessment (See section entitled “Validation of first field to first two fields HLA type imputation by HaploStats and Supplementary material”) |
| Bias | 9 | Described efforts to address potential sources of bias (See section entitled “Verification of Hardy-Weinberg Equilibrium (HWE) and generalizability of findings across Quebec HSCD”) |
| Study size | 10 | Explained how the study size was arrived at (See section entitled “Study design, ethics statement, data set, and HLA typings formats”) |
| Quantitative variables | 11 | Explained how quantitative variables were handled in the analyses. If applicable, describe which groupings were chosen and why (See section entitled “Validation of first field to first two fields HLA type imputation by HaploStats and Supplementary material”) |
| Statistical methods | 12 | (*a*) Described all statistical methods |
|  |  | (*b*) Explained how missing data were addressed |
|  |  | (*c*) Described analytical methods taking account of sampling strategy |
|  |  | (See sections entitled “Validation of first field to first two fields HLA type imputation by HaploStats and Supplementary material”, “Study design, ethics statement, data set, and HLA typings formats”, and “Verification of Hardy-Weinberg Equilibrium (HWE) and generalizability of findings across Quebec HSCD”, respectively) |
| Results | | |
| Participants | 13* | (a) Reported numbers of individuals at each stage of study—eg numbers potentially eligible, examined for eligibility, confirmed eligible, included in the study, completing follow-up, and analysed |
|  |  | (b) Gave reasons for non-participation at each stage |
|  |  | (c) Considering simplicity of study flow, a diagram was not deemed necessary |
| Descriptive data | 14* | (a) The only characteristic available in the Hema-Quebec registry that was required for our study was participants’ race. |
|  |  |  |
| Outcome data | 15* | Imputation performance characteristics are reported |
| Main results | 16 | (*a*) Imputation performance is reported |
|  |  |  |
|  |  |  |
| Other analyses | 17 | We report on analyses for verification of Hardy-Weinberg Equilibrium (HWE) and generalizability of findings across Quebec HSCD |
| Discussion | | |
| Key results | 18 | Summarised key results with reference to study objectives |
| Limitations | 19 | Limitations of the study, sources of potential bias or imprecision are discussed. |
| Interpretation | 20 | A cautious overall interpretation of results is provided, which considers objectives, limitations, analyses, and other relevant evidence |
| Generalisability | 21 | The generalisability of the study results is discussed |
| Other information | | |
| Funding | 22 | Source of funding is provided |
