# Supplementary material for: Performance of an allele‐level multi‐locus HLA genotype imputation tool in hematopoietic stem cell donors from Quebec
Source: Immun Inflamm Dis. 2017 Aug 25;5(4):551–9. doi: 10.1002/iid3.185 (PMC5691302; doi:10.1002/iid3.185)
Supplement: Supplementary file 2 — Supporting Information S1. [file IID3-5-551-s002.docx]

**Supplementary material 1: Imputation Accuracy and Genotype Ambiguity Measures**

*Imputation accuracy measures*

**Weighted city block distance**

The weighted city block distance ($d_{w}$*)* (1) introduced in materials and methods is defined as:

$d_{w}= \sum_{i=1}^{k} w_{i}|O_{i}-e_{i}|$, (1)

where *k* is the number of considered prediction ranges that contain the imputation probabilities, and $O_{i}$ is the observed proportion of correct imputations in the range $i$. The expected proportion of correct imputations in the interval $i$ rated $e_{i}$ is estimated by the average of imputation probabilities in that interval while the weight $w_{i}$ associated with each interval is estimated by the proportion of imputations in each range such that:

$w_{i}= \frac{x_{i}}{\sum_{i} x_{i}}$,

Where $x_{i}$ is the number of cases in the prediction range$i$.

**Recall**

Recall is defined as the fraction of correct HLA types (i.e. matching the true HLA types as determined by SBT) that were predicted by the imputation algorithm in the tested ambiguous dataset (2):

$Recall=\frac{\sum|correct imputations \cap all imputations |}{\sum|all imputations|}$ (2)

*Imputed genotype ambiguity measures*

**Typing resolution score**

The typing resolution score (TRS) is defined as follows:

$TRS=\sum_{g \in G} p^{2}\left( g \right)$ (3)

In this equation $p\left( g \right)$ represents the predicted probability of one of the possible unambiguous genotypes $g$ provided by the imputation procedure and $G$ represents the set of unambiguous genotypes. Low scores indicate highly ambiguous typing and high scores (up to 1) indicate little residual ambiguity. TRS can be measured at the level of phased or unphased multi-locus genotypes (HLA class I and class II), at the level of the individual loci and the individual HLA class (3).

When $A^{i}$ and $A^{j}$ represent an allele pair for a given locus, their associated predicted probability is obtained by summing all predicted probabilities of a haplotype pair generated for the given genotype as follows:

$\boldsymbol{p}\left( \boldsymbol{A}^{\boldsymbol{i}}\boldsymbol{,}\boldsymbol{A}^{\boldsymbol{j}} \right)\boldsymbol{=}\sum_{\boldsymbol{g \in G}} \boldsymbol{p}\left( \boldsymbol{g} \right)\boldsymbol{\cdot I(}\boldsymbol{A}^{\boldsymbol{i}}\boldsymbol{,}\boldsymbol{A}^{\boldsymbol{j}}\boldsymbol{)}$ (4)

where $I(A^{i},A^{j})$ is an indicator function that takes the value 1 if the imputed genotype $g$ contains the pair of alleles $A^{i}, A^{j}$. If the genotype doesn’t contain the pair of alleles $A^{i}, A^{j}$, the value of the indicator function is 0. TRS is then calculated, at the locus level, as described in equation (3) by replacing p(g) by p($A^{i}$,$A^{j}$).

Weighted city block distance, recall and TRS were calculated using R statistical software version 3.2.5.

**Supplementary material 2: Alleles (A) and Haplotypes (B) Frequency Distribution in the Héma-Quebec Dataset**

**(A)**

* **Freq**, Frequency

**(B)**

*Allele frequencies for the HLA-A, -B, -C, -DRB1 and -DQB1 loci (A) and haplotype (HLA-A~B~C~DRB1~DQB1) frequencies (B) in our Héma-Québec dataset were estimated by GENE[RATE] (4). Only haplotypes with a frequency ≥ 0.1% are listed. Note that for small samples sizes, haplotype frequencies maybe overestimated as a consequence of random sample fluctuation and truncation of the tail end of the frequency distribution that comprises the least frequent haplotypes (5).

**Supplementary material 3: Impact of Rare Alleles on the Performance of Allele-Level Multi-Locus Genotype Imputation by HaploStats using the Héma-Quebec Dataset**


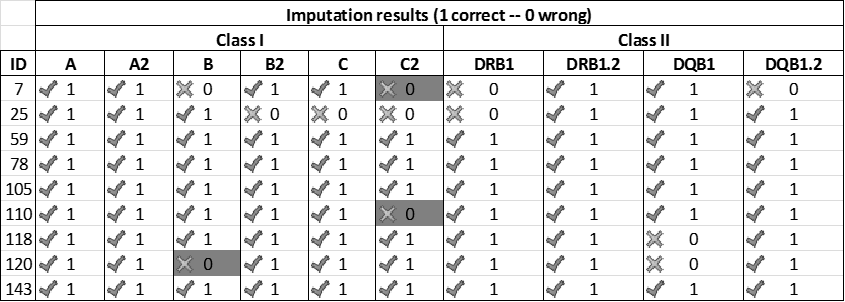


From a total of 144 self-reported Caucasian hematopoietic stem cell donors (HSCD) in the Héma-Quebec dataset, nine (6.25%) demonstrated rare alleles. This table shows the imputation results across HLA- class I and II loci for participants demonstrating ≥1 rare HLA alleles. Correct and wrong allele specification by the imputation process are represented by (√) and (X), respectively. Wrong imputations occurring in relation to rare alleles are marked by a grey background.

|  |
| --- |

**Supplementary material 4: Comparison of haplotypes in study sample with formerly observed haplotypes**

| **ID** | | **Sample Hap1** | **Sample Hap2** | **Sample haplotypes (Hap1/ Hap2) observed in** | | | | | | | | | | | | |
| --- | --- | --- | --- | --- | --- | --- | --- | --- | --- | --- | --- | --- | --- | --- | --- | --- |
|  |  |  |  | **US_CAU1** | **Freq** | **US_CAU2** | **Freq** | **US_oth1** | **US_oth2** | **FR1** | **Freq** | **FR2** | **Freq** | **AFN1*** | | **AFN2*** |
| 2 | | **A*24:02~B*35:03~C*04:01~DRB1*07:01~DQB1*02:02** | A*32:01~B*51:01~C*15:02~DRB1*15:01~DQB1*06:02 | NO | 0 | YES | 6.824988e-05 | NO | NO | NO | 0 | YES | 1.362170e-04 | NO | | NO |
| 2 | | **A*24:02~B*51:01~C*04:01~DRB1*07:01~DQB1*02:02** | **A*32:01~B*35:03~C*15:02~DRB1*15:01~DQB1*06:02** | NO | 0 | NO | 0 | NO | NO | NO | 0 | NO | 0 | NO | | NO |
| 2 | | **A*24:02~B*35:03~C*15:02~DRB1*07:01~DQB1*02:02** | **A*32:01~B*51:01~C*04:01~DRB1*15:01~DQB1*06:02** | NO | 0 | NO | 0 | NO | NO | NO | 0 | NO | 0 | NO | | NO |
| 2 | | **A*24:02~B*51:01~C*15:02~DRB1*07:01~DQB1*02:02** | A*32:01~B*35:03~C*04:01~DRB1*15:01~DQB1*06:02 | NO | 0 | YES | 2.656526e-05 | NO | NO | NO | 0 | YES | 5.262460e-05 | NO | | NO |
| 2 | | **A*24:02~B*35:03~C*04:01~DRB1*15:01~DQB1*02:02** | **A*32:01~B*51:01~C*15:02~DRB1*07:01~DQB1*06:02** | NO | 0 | NO | 0 | NO | NO | NO | 0 | NO | 0 | NO | | NO |
| 2 | | **A*24:02~B*51:01~C*04:01~DRB1*15:01~DQB1*02:02** | **A*32:01~B*35:03~C*15:02~DRB1*07:01~DQB1*06:02** | NO | 0 | NO | 0 | NO | NO | NO | 0 | NO | 0 | NO | | NO |
| 2 | | **A*24:02~B*35:03~C*15:02~DRB1*15:01~DQB1*02:02** | **A*32:01~B*51:01~C*04:01~DRB1*07:01~DQB1*06:02** | NO | 0 | NO | 0 | NO | NO | NO | 0 | NO | 0 | NO | | NO |
| 2 | | **A*24:02~B*51:01~C*15:02~DRB1*15:01~DQB1*02:02** | **A*32:01~B*35:03~C*04:01~DRB1*07:01~DQB1*06:02** | NO | 0 | NO | 0 | NO | NO | NO | 0 | NO | 0 | NO | | NO |
| 2 | | **A*24:02~B*35:03~C*04:01~DRB1*07:01~DQB1*06:02** | **A*32:01~B*51:01~C*15:02~DRB1*15:01~DQB1*02:02** | NO | 0 | NO | 0 | NO | NO | NO | 0 | NO | 0 | NO | | NO |
| 2 | | **A*24:02~B*51:01~C*04:01~DRB1*07:01~DQB1*06:02** | **A*32:01~B*35:03~C*15:02~DRB1*15:01~DQB1*02:02** | NO | 0 | NO | 0 | NO | NO | NO | 0 | NO | 0 | NO | | NO |
| 2 | | **A*24:02~B*35:03~C*15:02~DRB1*07:01~DQB1*06:02** | **A*32:01~B*51:01~C*04:01~DRB1*15:01~DQB1*02:02** | NO | 0 | NO | 0 | NO | NO | NO | 0 | NO | 0 | NO | | NO |
| 2 | | **A*24:02~B*51:01~C*15:02~DRB1*07:01~DQB1*06:02** | **A*32:01~B*35:03~C*04:01~DRB1*15:01~DQB1*02:02** | NO | 0 | NO | 0 | NO | NO | NO | 0 | NO | 0 | NO | | NO |
| 2 | | A*24:02~B*35:03~C*04:01~DRB1*15:01~DQB1*06:02 | **A*32:01~B*51:01~C*15:02~DRB1*07:01~DQB1*02:02** | YES | 1.483059e-04 | NO | 0 | NO | NO | YES | 1.892570e-04 | NO | 0 | NO | | NO |
| 2 | | A*24:02~B*51:01~C*04:01~DRB1*15:01~DQB1*06:02 | **A*32:01~B*35:03~C*15:02~DRB1*07:01~DQB1*02:02** | NO | 0 | NO | 0 | YES | NO | NO | 0 | NO | 0 | NO | | NO |
| 2 | | **A*24:02~B*35:03~C*15:02~DRB1*15:01~DQB1*06:02** | **A*32:01~B*51:01~C*04:01~DRB1*07:01~DQB1*02:02** | NO | 0 | NO | 0 | NO | NO | NO | 0 | NO | 0 | NO | | NO |
| 2 | | A*24:02~B*51:01~C*15:02~DRB1*15:01~DQB1*06:02 | **A*32:01~B*35:03~C*04:01~DRB1*07:01~DQB1*02:02** | YES | 9.387233e-05 | NO | 0 | NO | NO | YES | 9.919180e-05 | NO | 0 | NO | | NO |
| 23 | | A*03:01~B*35:01~C*04:01~DRB1*07:01~DQB1*02:02 | A*32:01~B*14:01~C*08:02~DRB1*11:01~DQB1*03:01 | NO | 0 | YES | 4.908935e-05 | NO | NO | NO | 0 | YES | 1.191100e-04 | NO | | NO |
| 23 | | A*03:01~B*14:01~C*08:02~DRB1*07:01~DQB1*02:02 | A*32:01~B*35:01~C*04:01~DRB1*11:01~DQB1*03:01 | NO | 0 | YES | 2.208123e-04 | NO | NO | NO | 0 | YES | 2.834820e-04 | NO | | NO |
| 23 | | A*03:01~B*35:01~C*04:01~DRB1*11:01~DQB1*03:01 | **A*32:01~B*14:01~C*08:02~DRB1*07:01~DQB1*02:02** | YES | 6.414496e-04 | NO | 0 | NO | NO | YES | 8.375580e-04 | NO | 0 | YES | | NO |
| 23 | | A*03:01~B*14:01~C*08:02~DRB1*11:01~DQB1*03:01 | A*32:01~B*35:01~C*04:01~DRB1*07:01~DQB1*02:02 | YES | 4.224118e-06 | NO | 0 | NO | NO | NO | 0 | NO | 0 | NO | | NO |
| 25 | | **A*01:01~B*15:03~C*02:10~DRB1*01:01~DQB1*03:01** | **A*68:01~B*15:18~C*07:04~DRB1*13:03~DQB1*05:01** | NO | 0 | NO | 0 | NO | NO | NO | 0 | NO | 0 | NO | | NO |
| 25 | | **A*01:01~B*15:18~C*02:10~DRB1*01:01~DQB1*03:01** | **A*68:01~B*15:03~C*07:04~DRB1*13:03~DQB1*05:01** | NO | 0 | NO | 0 | NO | NO | NO | 0 | NO | 0 | NO | | NO |
| 25 | | **A*01:01~B*15:03~C*07:04~DRB1*01:01~DQB1*03:01** | **A*68:01~B*15:18~C*02:10~DRB1*13:03~DQB1*05:01** | NO | 0 | NO | 0 | NO | NO | NO | 0 | NO | 0 | NO | | NO |
| 25 | | **A*01:01~B*15:18~C*07:04~DRB1*01:01~DQB1*03:01** | **A*68:01~B*15:03~C*02:10~DRB1*13:03~DQB1*05:01** | NO | 0 | NO | 0 | NO | NO | NO | 0 | NO | 0 | NO | | NO |
| 25 | | **A*01:01~B*15:03~C*02:10~DRB1*13:03~DQB1*03:01** | A*68:01~B*15:18~C*07:04~DRB1*01:01~DQB1*05:01 | NO | 0 | YES | 5.170630e-05 | NO | NO | NO | 0 | NO | 0 | NO | | NO |
| 25 | | **A*01:01~B*15:18~C*02:10~DRB1*13:03~DQB1*03:01** | **A*68:01~B*15:03~C*07:04~DRB1*01:01~DQB1*05:01** | NO | 0 | NO | 0 | NO | NO | NO | 0 | NO | 0 | NO | | NO |
| 25 | | **A*01:01~B*15:03~C*07:04~DRB1*13:03~DQB1*03:01** | **A*68:01~B*15:18~C*02:10~DRB1*01:01~DQB1*05:01** | NO | 0 | NO | 0 | NO | NO | NO | 0 | NO | 0 | NO | | NO |
| 25 | | **A*01:01~B*15:18~C*07:04~DRB1*13:03~DQB1*03:01** | **A*68:01~B*15:03~C*02:10~DRB1*01:01~DQB1*05:01** | NO | 0 | NO | 0 | NO | NO | NO | 0 | NO | 0 | NO | | NO |
| 25 | | **A*01:01~B*15:03~C*02:10~DRB1*01:01~DQB1*05:01** | A*68:01~B*15:18~C*07:04~DRB1*13:03~DQB1*03:01 | NO | 0 | YES | 6.252939e-07 | NO | NO | NO | 0 | NO | 0 | NO | | NO |
| 25 | | **A*01:01~B*15:18~C*02:10~DRB1*01:01~DQB1*05:01** | **A*68:01~B*15:03~C*07:04~DRB1*13:03~DQB1*03:01** | NO | 0 | NO | 0 | NO | NO | NO | 0 | NO | 0 | NO | | NO |
| 25 | | **A*01:01~B*15:03~C*07:04~DRB1*01:01~DQB1*05:01** | **A*68:01~B*15:18~C*02:10~DRB1*13:03~DQB1*03:01** | NO | 0 | NO | 0 | NO | NO | NO | 0 | NO | 0 | NO | | NO |
| 25 | | A*01:01~B*15:18~C*07:04~DRB1*01:01~DQB1*05:01 | **A*68:01~B*15:03~C*02:10~DRB1*13:03~DQB1*03:01** | YES | 1.685763e-05 | NO | 0 | NO | NO | NO | 0 | NO | 0 | YES | | NO |
| 25 | | **A*01:01~B*15:03~C*02:10~DRB1*13:03~DQB1*05:01** | **A*68:01~B*15:18~C*07:04~DRB1*01:01~DQB1*03:01** | NO | 0 | NO | 0 | NO | NO | NO | 0 | NO | 0 | NO | | NO |
| 25 | | **A*01:01~B*15:18~C*02:10~DRB1*13:03~DQB1*05:01** | **A*68:01~B*15:03~C*07:04~DRB1*01:01~DQB1*03:01** | NO | 0 | NO | 0 | NO | NO | NO | 0 | NO | 0 | NO | | NO |
| 25 | | **A*01:01~B*15:03~C*07:04~DRB1*13:03~DQB1*05:01** | **A*68:01~B*15:18~C*02:10~DRB1*01:01~DQB1*03:01** | NO | 0 | NO | 0 | NO | NO | NO | 0 | NO | 0 | NO | | NO |
| 25 | | **A*01:01~B*15:18~C*07:04~DRB1*13:03~DQB1*05:01** | **A*68:01~B*15:03~C*02:10~DRB1*01:01~DQB1*03:01** | NO | 0 | NO | 0 | NO | NO | NO | 0 | NO | 0 | NO | | NO |
| 30 | | **A*32:01~B*40:01~C*03:04~DRB1*04:04~DQB1*02:02** | **A*68:02~B*44:03~C*16:01~DRB1*07:01~DQB1*03:02** | NO | 0 | NO | 0 | NO | NO | NO | 0 | NO | 0 | NO | | NO |
| 30 | | **A*32:01~B*44:03~C*03:04~DRB1*04:04~DQB1*02:02** | **A*68:02~B*40:01~C*16:01~DRB1*07:01~DQB1*03:02** | NO | 0 | NO | 0 | NO | NO | NO | 0 | NO | 0 | NO | | NO |
| 30 | | **A*32:01~B*40:01~C*16:01~DRB1*04:04~DQB1*02:02** | **A*68:02~B*44:03~C*03:04~DRB1*07:01~DQB1*03:02** | NO | 0 | NO | 0 | NO | NO | NO | 0 | NO | 0 | NO | | NO |
| 30 | | **A*32:01~B*44:03~C*16:01~DRB1*04:04~DQB1*02:02** | **A*68:02~B*40:01~C*03:04~DRB1*07:01~DQB1*03:02** | NO | 0 | NO | 0 | NO | NO | NO | 0 | NO | 0 | NO | | NO |
| 30 | | **A*32:01~B*40:01~C*03:04~DRB1*07:01~DQB1*02:02** | **A*68:02~B*44:03~C*16:01~DRB1*04:04~DQB1*03:02** | NO | 0 | NO | 0 | NO | NO | NO | 0 | NO | 0 | NO | | NO |
| 30 | | **A*32:01~B*44:03~C*03:04~DRB1*07:01~DQB1*02:02** | **A*68:02~B*40:01~C*16:01~DRB1*04:04~DQB1*03:02** | NO | 0 | NO | 0 | NO | NO | NO | 0 | NO | 0 | NO | | NO |
| 30 | | **A*32:01~B*40:01~C*16:01~DRB1*07:01~DQB1*02:02** | **A*68:02~B*44:03~C*03:04~DRB1*04:04~DQB1*03:02** | NO | 0 | NO | 0 | NO | NO | NO | 0 | NO | 0 | NO | | NO |
| 30 | | A*32:01~B*44:03~C*16:01~DRB1*07:01~DQB1*02:02 | A*68:02~B*40:01~C*03:04~DRB1*04:04~DQB1*03:02 | NO | 0 | YES | 7.729153e-06 | NO | NO | NO | 0 | YES | 1.554060e-05 | NO | | NO |
| 30 | | A*32:01~B*40:01~C*03:04~DRB1*04:04~DQB1*03:02 | **A*68:02~B*44:03~C*16:01~DRB1*07:01~DQB1*02:02** | YES | 6.647439e-04 | NO | 0 | NO | NO | YES | 2.353800e-04 | NO | 0 | NO | | NO |
| 30 | | **A*32:01~B*44:03~C*03:04~DRB1*04:04~DQB1*03:02** | **A*68:02~B*40:01~C*16:01~DRB1*07:01~DQB1*02:02** | NO | 0 | NO | 0 | NO | NO | NO | 0 | NO | 0 | NO | | NO |
| 30 | | **A*32:01~B*40:01~C*16:01~DRB1*04:04~DQB1*03:02** | **A*68:02~B*44:03~C*03:04~DRB1*07:01~DQB1*02:02** | NO | 0 | NO | 0 | NO | NO | NO | 0 | NO | 0 | NO | | NO |
| 30 | | A*32:01~B*44:03~C*16:01~DRB1*04:04~DQB1*03:02 | **A*68:02~B*40:01~C*03:04~DRB1*07:01~DQB1*02:02** | YES | 3.507365e-06 | NO | 0 | NO | NO | NO | 0 | NO | 0 | NO | | NO |
| 30 | | **A*32:01~B*40:01~C*03:04~DRB1*07:01~DQB1*03:02** | **A*68:02~B*44:03~C*16:01~DRB1*04:04~DQB1*02:02** | NO | 0 | NO | 0 | NO | NO | NO | 0 | NO | 0 | NO | | NO |
| 30 | | **A*32:01~B*44:03~C*03:04~DRB1*07:01~DQB1*03:02** | **A*68:02~B*40:01~C*16:01~DRB1*04:04~DQB1*02:02** | NO | 0 | NO | 0 | NO | NO | NO | 0 | NO | 0 | NO | | NO |
| 30 | | **A*32:01~B*40:01~C*16:01~DRB1*07:01~DQB1*03:02** | **A*68:02~B*44:03~C*03:04~DRB1*04:04~DQB1*02:02** | NO | 0 | NO | 0 | NO | NO | NO | 0 | NO | 0 | NO | | NO |
| 30 | | **A*32:01~B*44:03~C*16:01~DRB1*07:01~DQB1*03:02** | **A*68:02~B*40:01~C*03:04~DRB1*04:04~DQB1*02:02** | NO | 0 | NO | 0 | NO | NO | NO | 0 | NO | 0 | NO | | NO |
| 42 | | A*01:01~B*37:01~C*06:02~DRB1*07:01~DQB1*02:02 | A*03:01~B*51:01~C*15:02~DRB1*11:01~DQB1*03:01 | NO | 0 | YES | 2.753719e-04 | NO | NO | NO | 0 | YES | 2.924250e-04 | NO | | YES |
| 42 | | A*01:01~B*37:01~C*15:02~DRB1*07:01~DQB1*02:02 | A*03:01~B*51:01~C*06:02~DRB1*11:01~DQB1*03:01 | NO | 0 | YES | 5.074501e-07 | NO | NO | NO | 0 | YES | 5.390000e-07 | NO | | NO |
| 42 | | A*01:01~B*51:01~C*15:02~DRB1*07:01~DQB1*02:02 | A*03:01~B*37:01~C*06:02~DRB1*11:01~DQB1*03:01 | NO | 0 | YES | 8.414007e-05 | NO | NO | NO | 0 | YES | 7.155810e-05 | NO | | NO |
| 42 | | A*01:01~B*37:01~C*06:02~DRB1*11:01~DQB1*03:01 | A*03:01~B*51:01~C*15:02~DRB1*07:01~DQB1*02:02 | YES | 5.198359e-04 | NO | 0 | NO | NO | YES | 5.225850e-04 | NO | 0 | YES | | NO |
| 42 | | A*01:01~B*51:01~C*06:02~DRB1*11:01~DQB1*03:01 | A*03:01~B*37:01~C*15:02~DRB1*07:01~DQB1*02:02 | YES | 1.458513e-06 | NO | 0 | NO | NO | YES | 4.470000e-06 | NO | 0 | NO | | NO |
| 42 | | A*01:01~B*51:01~C*15:02~DRB1*11:01~DQB1*03:01 | **A*03:01~B*37:01~C*06:02~DRB1*07:01~DQB1*02:02** | YES | 1.604042e-04 | NO | 0 | NO | NO | YES | 4.915700e-04 | NO | 0 | YES | | NO |
| 58 | | A*23:01~B*44:03~C*04:09~DRB1*07:01~DQB1*02:02 | A*29:02~B*44:03~C*16:01~DRB1*01:02~DQB1*05:01 | NO | 0 | YES | 2.636041e-05 | NO | NO | NO | 0 | YES | 1.200560e-04 | NO | | NO |
| 58 | | **A*23:01~B*44:03~C*04:09~DRB1*01:02~DQB1*05:01** | **A*29:02~B*44:03~C*16:01~DRB1*07:01~DQB1*02:02** | NO | 0 | NO | 0 | NO | NO | NO | 0 | NO | 0 | NO | | NO |
| 58 | | A*23:01~B*44:03~C*16:01~DRB1*01:02~DQB1*05:01 | A*29:02~B*44:03~C*04:09~DRB1*07:01~DQB1*02:02 | NO | 0 | NO | 0 | YES | NO | NO | 0 | NO | 0 | NO | | NO |
| 76 | | A*11:01~B*13:02~C*04:01~DRB1*07:01~DQB1*02:02 | A*30:01~B*53:01~C*06:02~DRB1*13:02~DQB1*06:04 | NO | 0 | YES | 6.701981e-06 | NO | NO | NO | 0 | YES | 4.020000e-06 | NO | | NO |
| 76 | | A*11:01~B*53:01~C*04:01~DRB1*07:01~DQB1*02:02 | A*30:01~B*13:02~C*06:02~DRB1*13:02~DQB1*06:04 | NO | 0 | YES | 1.198865e-04 | NO | NO | NO | 0 | YES | 7.076160e-05 | NO | | NO |
| 76 | | A*11:01~B*13:02~C*06:02~DRB1*07:01~DQB1*02:02 | A*30:01~B*53:01~C*04:01~DRB1*13:02~DQB1*06:04 | NO | 0 | YES | 2.045725e-05 | NO | NO | NO | 0 | YES | 1.226570e-05 | NO | | NO |
| 76 | | A*11:01~B*53:01~C*04:01~DRB1*13:02~DQB1*06:04 | **A*30:01~B*13:02~C*06:02~DRB1*07:01~DQB1*02:02** | YES | 1.111444e-04 | NO | 0 | NO | NO | YES | 1.711720e-04 | NO | 0 | NO | | NO |
| 76 | | A*11:01~B*13:02~C*06:02~DRB1*13:02~DQB1*06:04 | A*30:01~B*53:01~C*04:01~DRB1*07:01~DQB1*02:02 | YES | 1.339365e-05 | NO | 0 | NO | NO | NO | 0 | NO | 0 | NO | | NO |
| 82 | | A*01:01~B*08:01~C*03:04~DRB1*07:01~DQB1*02:02 | A*02:01~B*40:01~C*07:01~DRB1*15:01~DQB1*06:02 | NO | 0 | YES | 1.017182e-06 | NO | NO | NO | 0 | YES | 5.750000e-07 | NO | | NO |
| 82 | | A*01:01~B*40:01~C*03:04~DRB1*07:01~DQB1*02:02 | A*02:01~B*08:01~C*07:01~DRB1*15:01~DQB1*06:02 | NO | 0 | YES | 4.523861e-04 | NO | NO | NO | 0 | YES | 9.367610e-04 | NO | | YES |
| 82 | | A*01:01~B*08:01~C*07:01~DRB1*07:01~DQB1*02:02 | A*02:01~B*40:01~C*03:04~DRB1*15:01~DQB1*06:02 | NO | 0 | YES | 1.793051e-03 | NO | NO | NO | 0 | YES | 1.013142e-03 | NO | | YES |
| 82 | | A*01:01~B*40:01~C*07:01~DRB1*07:01~DQB1*02:02 | A*02:01~B*08:01~C*03:04~DRB1*15:01~DQB1*06:02 | NO | 0 | YES | 6.643694e-07 | NO | NO | NO | 0 | YES | 1.375720e-06 | NO | | NO |
| 82 | | A*01:01~B*40:01~C*03:04~DRB1*15:01~DQB1*06:02 | A*02:01~B*08:01~C*07:01~DRB1*07:01~DQB1*02:02 | YES | 1.529523e-04 | NO | 0 | NO | NO | YES | 1.310890e-04 | NO | 0 | NO | | NO |
| 82 | | A*01:01~B*08:01~C*07:01~DRB1*15:01~DQB1*06:02 | **A*02:01~B*40:01~C*03:04~DRB1*07:01~DQB1*02:02** | YES | 3.800719e-03 | NO | 0 | NO | NO | YES | 4.378508e-03 | NO | 0 | YES | | NO |
| 82 | | A*01:01~B*40:01~C*07:01~DRB1*15:01~DQB1*06:02 | A*02:01~B*08:01~C*03:04~DRB1*07:01~DQB1*02:02 | YES | 3.614169e-06 | NO | 0 | NO | NO | YES | 3.100000e-06 | NO | 0 | NO | | NO |
| 102 | | A*24:02~B*50:01~C*06:02~DRB1*07:01~DQB1*02:02 | A*29:02~B*39:06~C*07:02~DRB1*08:01~DQB1*04:02 | NO | 0 | YES | 7.492535e-06 | NO | NO | NO | 0 | YES | 1.446880e-05 | NO | | NO |
| 102 | | A*24:02~B*39:06~C*07:02~DRB1*07:01~DQB1*02:02 | A*29:02~B*50:01~C*06:02~DRB1*08:01~DQB1*04:02 | NO | 0 | YES | 6.676468e-06 | NO | NO | NO | 0 | NO | 0 | NO | | NO |
| 102 | | A*24:02~B*50:01~C*06:02~DRB1*08:01~DQB1*04:02 | A*29:02~B*39:06~C*07:02~DRB1*07:01~DQB1*02:02 | YES | 3.856581e-06 | NO | 0 | NO | NO | NO | 0 | NO | 0 | NO | | NO |
| 102 | | A*24:02~B*39:06~C*07:02~DRB1*08:01~DQB1*04:02 | **A*29:02~B*50:01~C*06:02~DRB1*07:01~DQB1*02:02** | YES | 1.011465e-03 | NO | 0 | NO | NO | YES | 1.578403e-03 | NO | 0 | YES | | NO |
| 114 | | **A*25:01~B*18:01~C*05:01~DRB1*07:01~DQB1*02:02** | **A*30:02~B*18:01~C*12:03~DRB1*10:01~DQB1*05:01** | NO | 0 | NO | 0 | NO | NO | NO | 0 | NO | 0 | NO | | NO |
| 114 | | **A*25:01~B*18:01~C*12:03~DRB1*07:01~DQB1*02:02** | A*30:02~B*18:01~C*05:01~DRB1*10:01~DQB1*05:01 | NO | 0 | YES | 1.977560e-05 | NO | NO | NO | 0 | YES | 1.725540e-05 | NO | | NO |
| 114 | | **A*25:01~B*18:01~C*05:01~DRB1*10:01~DQB1*02:02** | **A*30:02~B*18:01~C*12:03~DRB1*07:01~DQB1*05:01** | NO | 0 | NO | 0 | NO | NO | NO | 0 | NO | 0 | NO | | NO |
| 114 | | **A*25:01~B*18:01~C*12:03~DRB1*10:01~DQB1*02:02** | **A*30:02~B*18:01~C*05:01~DRB1*07:01~DQB1*05:01** | NO | 0 | NO | 0 | NO | NO | NO | 0 | NO | 0 | NO | | NO |
| 114 | | **A*25:01~B*18:01~C*05:01~DRB1*07:01~DQB1*05:01** | **A*30:02~B*18:01~C*12:03~DRB1*10:01~DQB1*02:02** | NO | 0 | NO | 0 | NO | NO | NO | 0 | NO | 0 | NO | | NO |
| 114 | | **A*25:01~B*18:01~C*12:03~DRB1*07:01~DQB1*05:01** | **A*30:02~B*18:01~C*05:01~DRB1*10:01~DQB1*02:02** | NO | 0 | NO | 0 | NO | NO | NO | 0 | NO | 0 | NO | | NO |
| 114 | | **A*25:01~B*18:01~C*05:01~DRB1*10:01~DQB1*05:01** | **A*30:02~B*18:01~C*12:03~DRB1*07:01~DQB1*02:02** | NO | 0 | NO | 0 | NO | NO | NO | 0 | NO | 0 | NO | | NO |
| 114 | | A*25:01~B*18:01~C*12:03~DRB1*10:01~DQB1*05:01 | **A*30:02~B*18:01~C*05:01~DRB1*07:01~DQB1*02:02** | YES | 4.059410e-05 | NO | 0 | NO | NO | YES | 2.690130e-05 | NO | 0 | NO | | NO |
| 118 | | **A*03:01~B*50:01~C*06:02~DRB1*07:01~DQB1*02:02** | A*68:02~B*57:02~C*18:01~DRB1*13:02~DQB1*05:01 | NO | 0 | YES | 4.710865e-06 | NO | NO | NO | 0 | NO | 0 | NO | | NO |
| 118 | | **A*03:01~B*57:02~C*06:02~DRB1*07:01~DQB1*02:02** | **A*68:02~B*50:01~C*18:01~DRB1*13:02~DQB1*05:01** | NO | 0 | NO | 0 | NO | NO | NO | 0 | NO | 0 | NO | | NO |
| 118 | | **A*03:01~B*50:01~C*18:01~DRB1*07:01~DQB1*02:02** | **A*68:02~B*57:02~C*06:02~DRB1*13:02~DQB1*05:01** | NO | 0 | NO | 0 | NO | NO | NO | 0 | NO | 0 | NO | | NO |
| 118 | | **A*03:01~B*57:02~C*18:01~DRB1*07:01~DQB1*02:02** | **A*68:02~B*50:01~C*06:02~DRB1*13:02~DQB1*05:01** | NO | 0 | NO | 0 | NO | NO | NO | 0 | NO | 0 | NO | | NO |
| 118 | | **A*03:01~B*50:01~C*06:02~DRB1*13:02~DQB1*02:02** | **A*68:02~B*57:02~C*18:01~DRB1*07:01~DQB1*05:01** | NO | 0 | NO | 0 | NO | NO | NO | 0 | NO | 0 | NO | | NO |
| 118 | | **A*03:01~B*57:02~C*06:02~DRB1*13:02~DQB1*02:02** | **A*68:02~B*50:01~C*18:01~DRB1*07:01~DQB1*05:01** | NO | 0 | NO | 0 | NO | NO | NO | 0 | NO | 0 | NO | | NO |
| 118 | | **A*03:01~B*50:01~C*18:01~DRB1*13:02~DQB1*02:02** | **A*68:02~B*57:02~C*06:02~DRB1*07:01~DQB1*05:01** | NO | 0 | NO | 0 | NO | NO | NO | 0 | NO | 0 | NO | | NO |
| 118 | | **A*03:01~B*57:02~C*18:01~DRB1*13:02~DQB1*02:02** | **A*68:02~B*50:01~C*06:02~DRB1*07:01~DQB1*05:01** | NO | 0 | NO | 0 | NO | NO | NO | 0 | NO | 0 | NO | | NO |
| 118 | | **A*03:01~B*50:01~C*06:02~DRB1*07:01~DQB1*05:01** | **A*68:02~B*57:02~C*18:01~DRB1*13:02~DQB1*02:02** | NO | 0 | NO | 0 | NO | NO | NO | 0 | NO | 0 | NO | | NO |
| 118 | | **A*03:01~B*57:02~C*06:02~DRB1*07:01~DQB1*05:01** | **A*68:02~B*50:01~C*18:01~DRB1*13:02~DQB1*02:02** | NO | 0 | NO | 0 | NO | NO | NO | 0 | NO | 0 | NO | | NO |
| 118 | | **A*03:01~B*50:01~C*18:01~DRB1*07:01~DQB1*05:01** | **A*68:02~B*57:02~C*06:02~DRB1*13:02~DQB1*02:02** | NO | 0 | NO | 0 | NO | NO | NO | 0 | NO | 0 | NO | | NO |
| 118 | | **A*03:01~B*57:02~C*18:01~DRB1*07:01~DQB1*05:01** | **A*68:02~B*50:01~C*06:02~DRB1*13:02~DQB1*02:02** | NO | 0 | NO | 0 | NO | NO | NO | 0 | NO | 0 | NO | | NO |
| 118 | | A*03:01~B*50:01~C*06:02~DRB1*13:02~DQB1*05:01 | **A*68:02~B*57:02~C*18:01~DRB1*07:01~DQB1*02:02** | NO | 0 | NO | 0 | YES | NO | NO | 0 | NO | 0 | NO | | NO |
| 118 | | **A*03:01~B*57:02~C*06:02~DRB1*13:02~DQB1*05:01** | **A*68:02~B*50:01~C*18:01~DRB1*07:01~DQB1*02:02** | NO | 0 | NO | 0 | NO | NO | NO | 0 | NO | 0 | NO | | NO |
| 118 | | **A*03:01~B*50:01~C*18:01~DRB1*13:02~DQB1*05:01** | **A*68:02~B*57:02~C*06:02~DRB1*07:01~DQB1*02:02** | NO | 0 | NO | 0 | NO | NO | NO | 0 | NO | 0 | NO | | NO |
| 118 | | A*03:01~B*57:02~C*18:01~DRB1*13:02~DQB1*05:01 | **A*68:02~B*50:01~C*06:02~DRB1*07:01~DQB1*02:02** | NO | 0 | NO | 0 | YES | NO | NO | 0 | NO | 0 | NO | | NO |
| 119 | | **A*30:01~B*13:02~C*06:02~DRB1*07:01~DQB1*02:02** | A*68:01~B*51:01~C*14:02~DRB1*01:01~DQB1*05:01 | NO | 0 | YES | 3.706027e-05 | NO | NO | NO | 0 | YES | 3.756720e-05 | NO | | NO |
| 119 | | A*30:01~B*51:01~C*14:02~DRB1*07:01~DQB1*02:02 | A*68:01~B*13:02~C*06:02~DRB1*01:01~DQB1*05:01 | NO | 0 | YES | 4.305122e-06 | NO | NO | NO | 0 | NO | 0 | NO | | NO |
| 119 | | A*30:01~B*13:02~C*06:02~DRB1*01:01~DQB1*05:01 | A*68:01~B*51:01~C*14:02~DRB1*07:01~DQB1*02:02 | YES | 2.340995e-04 | NO | 0 | NO | NO | YES | 2.126510e-04 | NO | 0 | NO | | NO |
| 119 | | A*30:01~B*51:01~C*14:02~DRB1*01:01~DQB1*05:01 | A*68:01~B*13:02~C*06:02~DRB1*07:01~DQB1*02:02 | YES | 2.486142e-06 | NO | 0 | NO | NO | YES | 4.912540e-06 | NO | 0 | NO | | NO |
| 120 | | A*24:02~B*15:24~C*03:03~DRB1*07:01~DQB1*02:02 | A*29:02~B*44:03~C*16:01~DRB1*16:01~DQB1*05:02 | NO | 0 | YES | 4.714986e-05 | NO | NO | NO | 0 | YES | 2.466550e-04 | NO | | NO |
| 120 | | A*24:02~B*44:03~C*16:01~DRB1*07:01~DQB1*02:02 | A*29:02~B*15:24~C*03:03~DRB1*16:01~DQB1*05:02 | NO | 0 | NO | 0 | NO | NO | NO | 0 | NO | 0 | YES | | NO |
| 120 | | A*24:02~B*15:24~C*03:03~DRB1*16:01~DQB1*05:02 | **A*29:02~B*44:03~C*16:01~DRB1*07:01~DQB1*02:02** | YES | 2.686345e-06 | NO | 0 | NO | NO | YES | 2.197120e-05 | NO | 0 | NO | | NO |
| 138 | | A*01:01~B*18:01~C*06:02~DRB1*11:01~DQB1*03:01 | A*02:01~B*57:01~C*08:02~DRB1*07:01~DQB1*03:03 | YES | 1.690020e-06 | NO | 0 | NO | NO | YES | 2.400000e-06 | NO | 0 | NO | | NO |
| 138 | | A*01:01~B*57:01~C*06:02~DRB1*11:01~DQB1*03:01 | A*02:01~B*18:01~C*08:02~DRB1*07:01~DQB1*03:03 | YES | 3.210495e-04 | NO | 0 | NO | NO | YES | 8.533570e-04 | NO | 0 | YES | | NO |
| 138 | | **A*01:01~B*18:01~C*08:02~DRB1*11:01~DQB1*03:01** | A*02:01~B*57:01~C*06:02~DRB1*07:01~DQB1*03:03 | NO | 0 | YES | 5.418004e-03 | NO | NO | NO | 0 | YES | 4.995937e-03 | NO | | YES |
| 138 | | A*01:01~B*57:01~C*06:02~DRB1*07:01~DQB1*03:03 | A*02:01~B*18:01~C*08:02~DRB1*11:01~DQB1*03:01 | YES | 8.091825e-03 | NO | 0 | NO | NO | YES | 7.053574e-03 | NO | 0 | YES | | NO |
| 138 | | A*01:01~B*18:01~C*08:02~DRB1*07:01~DQB1*03:03 | A*02:01~B*57:01~C*06:02~DRB1*11:01~DQB1*03:01 | NO | 0 | YES | 1.511684e-04 | NO | NO | NO | 0 | YES | 3.369090e-04 | NO | | NO |
| 138 | | A*01:01~B*57:01~C*08:02~DRB1*07:01~DQB1*03:03 | A*02:01~B*18:01~C*06:02~DRB1*11:01~DQB1*03:01 | NO | 0 | YES | 1.221866e-05 | NO | NO | NO | 0 | YES | 2.051600e-05 | NO | | NO |
| 139 | | **A*03:01~B*47:01~C*06:02~DRB1*07:01~DQB1*02:02** | **A*29:02~B*44:03~C*16:01~DRB1*07:01~DQB1*02:02** | NO | 0 | NO | 0 | NO | NO | NO | 0 | NO | 0 | NO | | NO |
| 139 | | A*03:01~B*44:03~C*16:01~DRB1*07:01~DQB1*02:02 | A*29:02~B*47:01~C*06:02~DRB1*07:01~DQB1*02:02 | NO | 0 | NO | 0 | NO | NO | NO | 0 | NO | 0 | YES | | NO |
| 140 | | A*01:01~B*08:01~C*03:03~DRB1*03:01~DQB1*02:01 | A*11:01~B*55:01~C*07:01~DRB1*07:01~DQB1*02:02 | YES | 2.428043e-06 | NO | 0 | NO | NO | YES | 1.940500e-06 | NO | 0 | NO | | NO |
| 140 | | A*01:01~B*55:01~C*03:03~DRB1*03:01~DQB1*02:01 | A*11:01~B*08:01~C*07:01~DRB1*07:01~DQB1*02:02 | YES | 2.443596e-05 | NO | 0 | NO | NO | NO | 0 | NO | 0 | NO | | NO |
| 140 | | A*01:01~B*08:01~C*07:01~DRB1*03:01~DQB1*02:01 | **A*11:01~B*55:01~C*03:03~DRB1*07:01~DQB1*02:02** | YES | 5.981507e-02 | NO | 0 | NO | NO | YES | 4.780452e-02 | NO | 0 | YES | | NO |
| 140 | | A*01:01~B*55:01~C*03:03~DRB1*07:01~DQB1*02:01 | A*11:01~B*08:01~C*07:01~DRB1*03:01~DQB1*02:02 | YES | 3.910317e-05 | NO | 0 | NO | NO | YES | 5.802830e-05 | NO | 0 | YES | | NO |
| 140 | | A*01:01~B*08:01~C*07:01~DRB1*07:01~DQB1*02:01 | A*11:01~B*55:01~C*03:03~DRB1*03:01~DQB1*02:02 | YES | 1.242573e-03 | NO | 0 | NO | NO | YES | 1.523156e-03 | NO | 0 | YES | | NO |
| 140 | | A*01:01~B*55:01~C*03:03~DRB1*03:01~DQB1*02:02 | A*11:01~B*08:01~C*07:01~DRB1*07:01~DQB1*02:01 | NO | 0 | YES | 3.301709e-05 | NO | NO | NO | 0 | YES | 2.386290e-05 | NO | | NO |
| 140 | | A*01:01~B*08:01~C*07:01~DRB1*03:01~DQB1*02:02 | A*11:01~B*55:01~C*03:03~DRB1*07:01~DQB1*02:01 | NO | 0 | YES | 1.655194e-04 | NO | NO | NO | 0 | YES | 2.807360e-04 | NO | | YES |
| 140 | | A*01:01~B*55:01~C*03:03~DRB1*07:01~DQB1*02:02 | A*11:01~B*08:01~C*07:01~DRB1*03:01~DQB1*02:01 | NO | 0 | YES | 1.271295e-03 | NO | NO | NO | 0 | YES | 7.609360e-04 | NO | | YES |
| 140 | | A*01:01~B*08:01~C*07:01~DRB1*07:01~DQB1*02:02 | A*11:01~B*55:01~C*03:03~DRB1*03:01~DQB1*02:01 | NO | 0 | YES | 6.940816e-05 | NO | NO | NO | 0 | YES | 1.425860e-04 | NO | | NO |
| 140 | | A*01:01~B*55:01~C*07:01~DRB1*07:01~DQB1*02:02 | A*11:01~B*08:01~C*03:03~DRB1*03:01~DQB1*02:01 | NO | 0 | YES | 2.160998e-06 | NO | NO | NO | 0 | YES | 1.290000e-06 | NO | | NO |
| 142 | | A*26:01~B*07:02~C*07:02~DRB1*15:01~DQB1*06:02 | **A*33:01~B*40:01~C*14:02~DRB1*15:01~DQB1*06:02** | YES | 9.901020e-04 | NO | 0 | NO | NO | YES | 1.027636e-03 | NO | 0 | NO | | NO |
| 142 | | A*26:01~B*40:01~C*07:02~DRB1*15:01~DQB1*06:02 | A*33:01~B*07:02~C*14:02~DRB1*15:01~DQB1*06:02 | NO | 0 | NO | 0 | YES | NO | NO | 0 | NO | 0 | NO | | NO |
| 142 | | A*26:01~B*40:01~C*14:02~DRB1*15:01~DQB1*06:02 | A*33:01~B*07:02~C*07:02~DRB1*15:01~DQB1*06:02 | NO | 0 | YES | 3.519238e-05 | NO | NO | NO | 0 | YES | 5.481020e-05 | NO | | NO |
|  | | | | | | | | | | | | | |  |  |  |

**√** probabiity of phased genotype

***** frequencies reported at 1e-2

**US_CAU**, Haplotypes observed among self-reported Caucasians in the US-Registry (NMDP-2011)

**US_oth**, Haplotypes observed self-reported non-Caucasians in the US-Registry (NMDP-2011)

**FR**, Haplotypes observed in RFGM (Registre France Greffe Moelle)

**AFN**, Haplotypes observed in the allele frequency website http://www.allelefrequencies.net/)

**N.B**, Possible new haplotypes are highlighted in bold

**Hap**, Haplotype

**Freq**, Frequency

**REFERENCES**

1. Merigó JM. CM. Decision-making with distance measures and induced aggregation operators. *Computers & Industrial Engineering*. 2011;**60** 66–76.

2. Madbouly A, Gragert L, Freeman J, et al. Validation of statistical imputation of allele-level multilocus phased genotypes from ambiguous HLA assignments. *Tissue Antigens*. 2014;**84**:285-92.

3. Paunic V, Gragert L, Schneider J, Muller C, Maiers M. Charting improvements in US registry HLA typing ambiguity using a typing resolution score. *Hum Immunol*. 2016;**77**:542-9.

4. Nunes JM. Using uniformat and gene[rate] to Analyze Data with Ambiguities in Population Genetics. *Evol Bioinform Online*. 2015;**11**:19-26.

5. Pappas DJ, Tomich A, Garnier F, Marry E, Gourraud PA. Comparison of high-resolution human leukocyte antigen haplotype frequencies in different ethnic groups: Consequences of sampling fluctuation and haplotype frequency distribution tail truncation. *Hum Immunol*. 2015;**76**:374-80.
